# Distinct cuproptosis patterns in hepatocellular carcinoma patients correlate with unique immune microenvironment characteristics and cell-cell communication, contributing to varied overall survival outcomes

**DOI:** 10.3389/fimmu.2024.1379690

**Published:** 2024-05-28

**Authors:** Yanhong Wang, Xinyu Mang, Xiaohong Guo, Junfeng Pu

**Affiliations:** ^1^ Shanghai Fourth People’s Hospital, and School of Medicine, Tongji University, Shanghai, China; ^2^ Department of Biochemistry and Molecular Biology, State Key Laboratory of Common Mechanism Research for Major Diseases, Institute of Basic Medical Sciences Chinese Academy of Medical Sciences, School of Basic Medicine Peking Union Medical College, Beijing, China; ^3^ Department of Pharmacy, People's Hospital of Gansu Province, Lanzhou, Gansu, China

**Keywords:** hepatocellular carcinoma, cuproptosis, TME, prognosis, scRNA-seq, cell-cell communication

## Abstract

**Background:**

Hepatocellular carcinoma (HCC), a prevalent cancer, is linked to cuproptosis in tumor progression. However, cuproptosis's impact on HCC prognosis and its role in the tumor microenvironment remain unclear. We aimed to explore the correlation between cellular cuproptosis and the immune microenvironment in HCC, providing potential immunotherapeutic insights.

**Methods:**

Examining cuproptosis-related genes and the immune microenvironment through consensus clustering and WGCNA. Risk models were constructed using LASSO Cox analysis and validated in an independent cohort. Gene expression data from The Cancer Genome Atlas (TCGA) database and single-cell RNA sequencing (scRNA-seq) data from the Gene Expression Omnibus (GEO) database were utilized. We scored cuproptosis expression and explored immunoinfiltration and cell-cell communication. Differential signals in T_memory cells were compared across different cuproptosis levels.

**Results:**

Cuproptosis genes associated with fibroblast recruitment (GLS) and macrophage infiltration (FDX1). Liver cancer patients categorized into two subtypes based on cuproptosis gene expression. High expression of DLAT, GLS, and CDKN2A linked to immunosuppression (TGF-β), while high FDX1, MTF1, LIAS, and LIPT1 expression enhanced communication with non-immune cells. Developed reliable prognostic signature score and nomogram using cuproptosis-related genes. Single-cell analysis revealed differences in T_memory and TAM infiltration based on cuproptosis scores, with SPP1 and MIF as dominant signaling molecules. Finally, the results of *in vitro* experiments showed that when DLAT or CDKN2A was knocked down, the proliferation, migration, and invasion of HCC cells were significantly decreased.

**Conclusion:**

Our study demonstrates that cuproptosis affects the immune microenvironment and cell-cell communication. Identified 9 genetic markers predicting survival outcomes and immunotherapy responses. Evaluating cuproptosis signaling can optimize immunotherapeutic strategies for hepatocellular carcinoma.

## 1 Introduction

Hepatocellular carcinoma (HCC) is a prevalent form of primary liver cancer (1). Surgical interventions like hepatectomy or liver transplantation are typically employed as curative measures, complemented by percutaneous ablation, radiotherapy, transarterial therapy, and systemic therapy options (2), including immunotherapy (3). Despite these treatment modalities, the 5-year survival rate remains low at 21%, and the mortality rate has shown a distressing increase in recent decades (4).The lack of specific biomarkers is one of the important reasons for the very poor prognosis of most patients (5). Therefore, identifying novel prognostic biomarkers is crucial to enhance survival rates in liver cancer patients.

Regulatory cell death (RCD), also referred to as programmed cell death (PCD), plays a pivotal role in tumorigenesis (6). Copper ions are vital for maintaining normal cellular function and homeostasis. However, an accumulation of copper ions can induce cellular damage (7). Evidence suggests that copper signaling is associated with cell proliferation, tumor growth, and metastasis in cancer (8). Previous studies have identified cell death induced by copper ions through protein lipid acylation, which is called cuproptosis (9). Nevertheless, the precise biological mechanisms underlying cuproptosis and its potential as an indicator for evaluating HCC remain poorly understood.

The tumor immune microenvironment (TME) plays a significant role in HCC development and patient prognosis (10). The tumor immune microenvironment (TME) is a dynamic system composed of multiple cell types and complex cellular components (10, 11). Furthermore, TME-based immunotherapy has shown promise in treating hepatocellular carcinoma (12). Evidence suggests that regulatory cell death (RCD) plays a significant role in tumor immunity and the tumor microenvironment (TME), thereby affecting the efficacy of immunotherapy (13). However, the relationship between cuproptosis and TME in hepatocellular carcinoma remains unclear. Therefore, this study systematically investigated the association between cuproptosis and TME in liver cancer, utilizing data from The Cancer Genome Atlas (TCGA). The findings revealed that cuproptosis was linked to an active immune microenvironment characterized by strong immunogenicity.

The utilization of single-cell RNA sequencing technology has greatly contributed to our understanding of cancer development, particularly in unraveling the immune microenvironment of cancer and identifying potential immunotherapeutic targets (10, 14, 15). In this study, we further explored the connection between cuproptosis and the immune microenvironment by analyzing single-cell transcriptome data. We compared the differences in cell communication, specifically focusing on T_memory cells. The results further confirmed the impact of cuproptosis on the immune microenvironment of liver cancer, highlighting its potential as a marker for immunotherapy.

## 2 Method

### 2.1 Data sources

To conduct our analysis, we obtained the gene expression, mutation data, and clinical information from the TCGA-LIHC project via the UCSC Xena platform (http://xena.ucsc.edu). We included a total of 377 patients, and for the follow-up analysis, we also selected 50 normal individuals. Additionally, we acquired a single-cell RNA sequencing dataset (accession number: GSE149614) consisting of 10 samples and 34,414 cells from the GEO database to validate the immune characteristics of hepatocellular carcinoma.

### 2.2 Immune infiltration analysis

For immune infiltration analysis, we employed the ESTIMATE algorithm (16) to evaluate the ratio of stromal and immune cells in the tumor microenvironment as well as tumor purity. Furthermore, we utilized EPIC algorithms (17) to compute the proportions of immune cells in each tumor microenvironment. Additionally, we downloaded the activation levels of the seven-step anti-tumor immune cycle from the TIP database (18).

### 2.3 Subtyping and differential gene analysis

To subtype the liver cancer patients and perform differential gene analysis, we employed a consensus clustering algorithm based on the expression of cuproptosis-related genes. This approach classified the patients into two subtypes: Group A (high expression of DLAT, GLS, and CDKN2A genes) and Group B (high expression of PDHA1, FDX1, MTF1, LIAS, and LIPT1 genes). We repeated the hierarchical clustering 500 times, each time selecting 80% of the samples. Finally, we determined the optimal number of clusters based on the cumulative distribution function and selected stable clustering results.

To screen for differentially expressed genes in both isoforms, we used the R package DESeq2 (19) with a significance threshold of |log2FoldChange| > 1 and an adjusted P-value < 0.05. Subsequently, the differential genes were subjected to gene ontology (GO) functional enrichment analysis using the R package ClusterProfiler (20). We also performed functional analysis based on the KEGG database using the GSEA algorithm. The ssGSEA (21) method was employed to measure the activity of cancer markers in tumor samples, and the enrichment scoring method was set as “singscore.” Furthermore, we analyzed the difference in marker activity between the two subtypes using the Wilcox test.

### 2.4 Weighted gene co-expression network analysis

We conducted co-expression network analysis of the differential genes using the WGCNA package in R. For this analysis, we set 4 as the soft threshold. Through clustering analysis, we identified highly similar modules. We then assessed the correlation between these modules and various factors such as stromal score, immune score, tumor purity, and different immune cell ratios. To further investigate the role of methylation, we examined the methylation patterns in the UALCAN database (22). This allowed us to identify key genes associated with methylation. Specifically, we focused on genes that exhibited higher methylation levels in the tumor group compared to the normal group.

### 2.5 Construction of risk scores

To ascertain the predictive capabilities of our model, we randomly divided the TCGA dataset into a training and test set. In the training set, we utilized the glmnet R package to construct a LASSO Cox regression model, employing the key genes identified earlier. This model was then evaluated in the test set to assess its validity. Next, we employed the median risk score as a threshold to partition the patients into high and low-risk groups. The Kaplan-Meier method was employed to generate survival curves for these groups, and the log-rank test was utilized to determine any significant differences in survival. Additionally, we calculated the time-dependent AUC by utilizing the R package timeROC (23) to evaluate the accuracy of our predictions. To further visualize the prognostic value of our model, we employed the R package rms to create a nomogram that incorporates pooled clinical variables. We employed calibration curves to assess the concordance between the predicted and observed risks indicated by the nomogram. Moreover, decision curve analysis was performed to measure the net benefit (NB) of utilizing the nomogram as a screening tool to determine if interventions are warranted for truly high-risk patients.

### 2.6 Mutation analysis

The R package maftools was used to analyze the mutation data obtained from patients with liver cancer. By employing waterfall plots, we visualized the mutation profile of the patients. Additionally, we calculated the tumor mutation load (TMB) by determining the total number of mutations present in each patient. To assess the mutation differences between the two groups of patients, we conducted the Fisher test.

### 2.7 scRNA-seq data clustering and dimensionality reduction

The Seurat (24) package was used to perform unsupervised clustering of individual cells, and we performed principal component analysis using the top 2000 high variance features in the dataset and downstream analysis using the top 20 principal components. Cell subclusters were identified using the “FindClusters” function (resolution = 0.6) and visualized using Uniform Manifold Approximation and Projection (UMAP).

### 2.8 Cuproptosis score calculation and cell-cell communication analysis

The irGSEA R package is used to calculate cuproptosis associated gene scores, obtained using the singscore algorithm. The scores of all cells in the samples were averaged, and all samples were divided into groups with high expression and low expression of cuproptosis related genes in a ratio of 1:1.

The R package Cellchat (25) was used to analyze cell-cell communication in single cell data and to compare the differences in cell-cell communication between the high and low expression groups of hepatocytes.

### 2.9 Cell culture and transfection

Human liver cancer cells HepG2 and SMMC7721 were procured from the Chinese Cell Bank (Shanghai, China). They were cultured in Dulbecco’s modified Eagle’s medium (DMEM, Gibco, NY, USA) supplemented with 10% fetal bovine serum and 100 U/ml penicillin-streptomycin, maintained at 37°C with 5% CO_2_. siRNAs targeting DLAT and CDKN2A, along with their respective negative control (NC) sequences, were obtained from RiboBio Co., Ltd. (Guangzhou, China). Cell transfections were carried out using Lipofectamine 3000 reagents (Invitrogen, Grand Island, NY, USA) in accordance with the manufacturer’s guidelines. After a 48-hour incubation period with Lipofectamine 3000, the HepG2 and SMMC7721 cells were harvested and utilized for the pertinent experiments. The sequences of siRNAs targeting DLAT were as follows: 5’-CAGTGAATTGTCTTTTAGACAAC-3’; siCDKN2A: 5’-GAUGCUUCGUCUACGAGAATT-3’; si-NC: Ribobio, # siN0000001-1.

### 2.10 Western blot assay

The HepG2 and SMMC7721 cells were harvested and rinsed with ice-cold PBS solution for the Western blot assay. Subsequently, cell lysis was carried out in RIPA cell lysis buffer (Solaibao, Beijing, China), supplemented with complete™ Protease Inhibitor Cocktail. Protein concentrations in the resulting supernatant were determined using the bicinchoninic acid (BCA) Assay (Solaibao, Beijing, China). The protein extracts were resolved on 10% SDS-PAGE gels and subsequently transferred onto PVDF membranes (Immobilon-P, CA, USA). After blocking with 5% skimmed milk, these membranes were subjected to overnight incubation at 4°C with DLAT (Cat#: 12362, Cell Signaling Technology, USA) or CDKN2A (Cat#: 18769, Cell Signaling Technology, USA) antibodies. Following three washes in TBST, the membranes were exposed to the secondary antibody at room temperature for 1 hour. Signal detection was accomplished using an enhanced chemiluminescence (ECL) kit (Immobilon-P, CA, USA), and band visualization was facilitated by the ChemiDoc™ Touch Imaging System (Bio-Rad). Relative quantification was performed by utilizing β-actin as an internal reference for normalization.

### 2.11 Cell counting kit−8

Cell proliferation capacity was assessed using the Cell Counting Kit‐8 purchased from Dojindo Laboratories (Japan). Initially, cells were plated at a density of 3×10^3^ cells/well in 100 μL of medium within 96‐well microplates (Corning, NY, USA). Following 24 hours of incubation, 10 μL of CCK‐8 reagent was added into each well and allowed to incubate for 1.5 h. Subsequently, the absorbance was measured at 450 nm using a microplate reader (Molecular Devices, Sunnyvale, USA). Cellular proliferation was quantified based on the absorbance readings.

### 2.12 Colony formation assay

The HepG2 and SMMC7721 cells were collected, resuspended in complete medium, and then inoculated into the wells at a density of 2000 cells per dish. The culture medium was changed periodically until visible colonies formed. Finally, the colonies were fixed with methanol and stained with a crystal violet solution (Beyotime Biotechnology, China). This experiment was repeated three times.

### 2.13 Migration and invasion assay

Transwell assays were conducted utilizing 8-μm pore transwell compartments (Cat. No. PTEP24H48, Millipore, Billerica, USA). In the migration assay, 200 µL of HepG2 and SMMC7721 cells (1×10^5^) were placed in the upper chamber with serum-free medium, while the lower chamber held 800 µL of DMEM supplemented with 10% FBS. For invasion assay, Matrigel (BD Biosciences, USA) was diluted in serum-free medium at a ratio of 1:19, then added to each well, and 1×10^5^ cells were suspended in the upper compartment. The subsequent steps mirrored those of the migration assay. Following incubation at 37°C for 24 h, the medium was discarded, and cells that had invaded the lower surface of the membrane were fixed in methanol for 30 minutes. Staining was performed using crystal violet (Beyotime Biotechnology, China) for 30 minutes at room temperature. The migrated and invaded cells were counted in 5 separate visual fields at ×20 magnification using an Olympus microscope.

### 2.14 RNA extraction and quantitative real time polymerase chain reaction

The SMMC7721 and HepG2 cells were seeded in 6-well plates at a density of 2 × 10^5^ cells/well and treated using Elesclomol 200 nM for 24 h. The total RNA was extracted using Trizol reagent (Invitrogen, Carlsbad, CA). The cDNA synthesis was performed using RevertAid First Strand cDNA Synthesis kit (Thermo Fisher Scientific). The PowerUp SYBR Green Master Mix (Applied Biosystems) was used for qRT-PCR on an FTC-3000p Real-Time PCR System (Funglyn Biotech, China). The relative gene expression was determined using the 2^-ΔΔCT^ method with actin as the reference gene. The primer sequences are for *BCAT1*: forward, 5′-GTGGAGTGGTCCTCAGAGTTT-3′ and reverse, 5′-AGCCAGGGTGCAATGACAG-3; for *TLR8*: forward, 5′-ATGTTCCTTCAGTCGTCAATGC-3′ and reverse, 5′-TTGCTGCACTCTGCAATAACT-3; for *IL4I1*: forward, 5′-TGATGTCCGAGGATGGCTTCT-3′ and reverse, 5′-TGTACTGGAGTCTGTCGCTGA-3; for *CASP5*: forward, 5′-TCACCTGCCTGCAAGGAATG-3′ and reverse, 5′-TCTTTTCGTCAACCACAGTGTAG-3; for *FCRL5*: forward, 5′-ATGTTCCTTCAGTCGTCAATGC-3′ and reverse, 5′-CCTCAAGGATATTGTCTGGGGTT-3; for *CCR8*: forward, 5′-CTGTCTGACCTGCTTTTTGTCT-3′ and reverse, 5′-CCACTTTGCACATTACAGTCCC-3; for *PDCD1*: forward, 5′-CCAGGATGGTTCTTAGACTCCC-3′ and reverse, 5′-TTTAGCACGAAGCTCTCCGAT-3; for *IFI30*: forward, 5′-CCCCTCTGCAAGCGTTAGAC-3′ and reverse, 5′-CCCGCAGGTATAGATTGCCT-3; for *MMP12*: forward, 5′-GATCCAAAGGCCGTAATGTTCC-3′ and reverse, 5′-TGAATGCCACGTATGTCATCAG-3; for *Actin*: forward, 5′-TCCCTGGAGAAGAGCTACGA-3′ and reverse, 5′-TACAGGTCTTTGCGGATGTC-3; The cells without Elesclomol treatment is selected as control.

### 2.15 Statistical analysis

The above statistics and analysis were performed using R (v 4.1.3) software. Figures were stitched together by Adobe Illustrator software. Comparative analysis of differences in box plots was performed using Wilcoxon rank sum test. Spielman’s coefficient was used for correlation analysis. The chi-square test (Fisher exact test if necessary) was used for comparison of clinical characteristics between the two groups. Multifactorial logistic regression analysis was used to assess the clinical characteristics affecting clustering. Kaplan Meier method was used to plot survival curves. Cox analysis was used to assess the characteristics associated with survival. All hypothesis tests were two-sided, and p-values for multiple tests were corrected by the FDR method, and corrected *P*-values <0.05 were considered significant.

## 3 Results

### 3.1 Differential cuproptosis-associated gene expression in hepatocellular carcinoma drives distinct prognoses among subtypes, linked to the immune microenvironment

**Figure 1:**
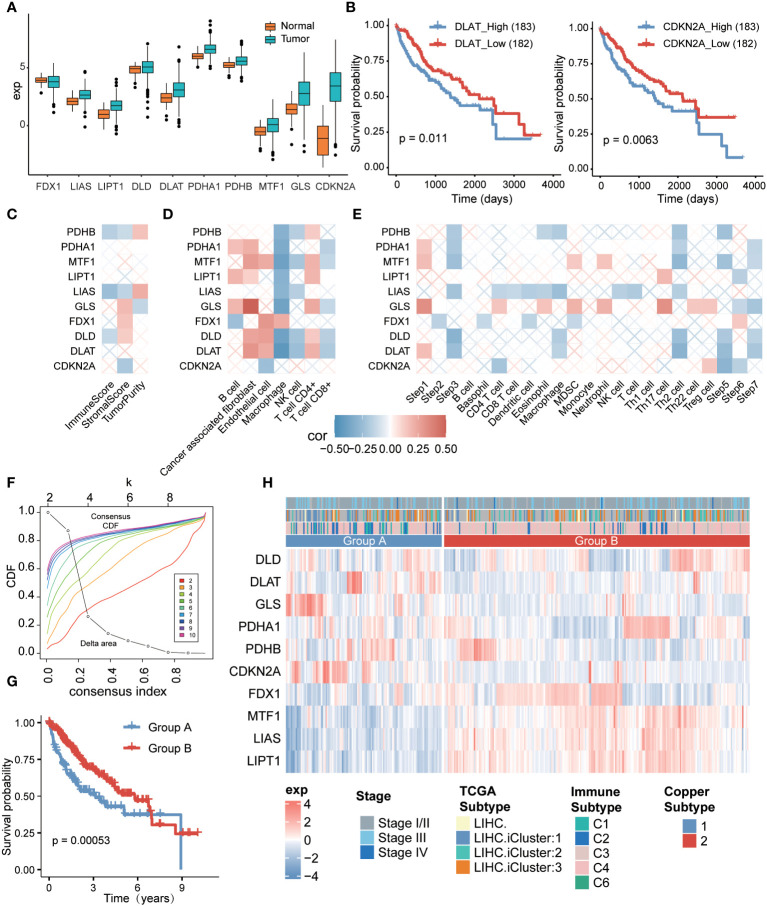
Relationship between cuproptosis-related genes and tumor microenvironment (TME) in hepatocellular carcinoma and cuproptosis typing. **(A)** Expression of cuproptosis-related genes between the tumor group and the normal group in hepatocellular carcinoma. **(B)** Survival analysis based on high and low expression of DLAT (left) and CDKN2A (right) in hepatocellular carcinoma. **(C)** Relationship between cuproptosis-associated genes and immune scores based on ESTIMATE method. **(D)** Relationship between cuproptosis-associated genes, immune score, and immune cell infiltration rate based on EPIC method. **(E)** Relationship between cuproptosis-associated genes and immune cycle activity based on the TIP method. **(F)** Consensus clustering based on cuproptosis-associated gene expression. **(G)** Comparison of overall survival rates between the two cuproptosis subtypes. **(H)** Heatmap displaying gene expression patterns in samples from the two subtypes, with top annotations indicating patient stage, TCGA subtype, and immune subtype.

To investigate the relationship between cuproptosis and hepatocellular carcinoma (HCC), we analyzed the differential profile of 10 cuproptosis-related genes (9) in tumor versus normal tissues. Our findings revealed that these cuproptosis-related genes were expressed at higher levels in tumor tissues compared to normal tissues (**Figure 1A**). Subsequently, we categorized liver cancer patients into two groups based on the median expression of these genes, separating them into high and low expression groups. By analyzing the prognostic differences between these groups, we observed that patients with low expression of DLAT and CDKN2A had significantly better prognosis compared to those with high expression (**Figure 1B**). These results indicated a close relationship between hepatocellular carcinoma and the expression levels of cuproptosis-related genes.

To further explore the connection between cuproptosis-related genes and the tumor immune microenvironment, we employed the ESTIMATE algorithm. The analysis demonstrated significant positive correlations of PDHB and LIAS with tumor purity, accompanied by negative correlations with immune score and interstitial score (**Figure 1C**). Additionally, we employed the EPIC method to assess immune cell infiltration associated with cuproptosis. The results indicated positive correlations between cuproptosis-related genes and various non-immune cells, particularly fibroblasts (**Supplementary Figure S1A**) and CD4^+^ T cells. Among macrophages, only FDX1 displayed a positive correlation (**Figure 1D**, **Supplementary Figure S1B**), while the remaining nine cuproptosis-related genes exhibited significant negative correlations. As an example, we can consider the gene GLS (**Figure 1D**, **Supplementary Figure S1C**). Moreover, we performed an analysis of the anti-cancer immune status and tumor-infiltrating immune cells, investigating the seven-step cancer-immunity cycle using the TIP method specifically for cuproptosis-related genes. We observed that the majority of genes were expressed in the initial step of the cancer-immunity cycle, which involves the release of antigens from cancer cells. Specifically, LIPT1 and LIAS displayed negative correlations with the recruitment of various immune cells (**Figure 1E**), whereas GLS was involved in multiple steps of the cycle. These findings indicate a strong association between the regulation of cuproptosis and the tumor immune microenvironment in hepatocellular carcinoma.

Subsequently, we conducted an analysis of cuproptosis subtypes in hepatocellular carcinoma based on the expression of cuproptosis-related genes, which exhibited significant correlations with each other (**Supplementary Figure S1D**). Employing consensus clustering, we grouped the hepatocellular carcinoma patients based on the expression patterns of cuproptosis-related genes. With a chosen consistency matrix k value of 2 (**Figure 1F**), the patients were divided into two distinct groups, indicating different cuproptosis-related gene expression profiles. Notably, patients in group B demonstrated a longer overall survival compared to those in group A (*P*=0.00053, **Figure 1G**). Specifically, genes DLAT, GLS, and CDKN2A were significantly overexpressed in group A, while genes PDHA1, FDX1, MTF1, LIAS, and LIPT1 were significantly overexpressed in group B (**Figure 1H**). Furthermore, we identified a significant correlation between the cuproptosis subtypes and the immune subtypes (26) (*P*=9.12e-07). However, the correlation with the TCGA classification (*P*=0.1071) was not statistically significant (**Figure 1H**, **Supplementary Table 1**), suggesting that the immune response subtype was more prevalent among patients in group B.

We conducted further analysis to examine the variations in the immune microenvironment between the two subtypes. The findings revealed higher levels of proliferation, wound healing, macrophage regulation, and lymphocyte infiltration in group A. Particularly, there was a notable elevation in the transforming growth factor-β response in group A, indicating a strong immunosuppressive effect (**Supplementary Figure S1E**). Additionally, group A exhibited higher levels of macrophage regulation, primarily observed in M0 macrophages. On the other hand, the functions of group B were more related to communication with non-immune cells and cell maintenance (**Supplementary Figures S1F**, **S2E**, **S3**).

These results indicate that the upregulation of cuproptosis genes, such as DLAT, GLS, and CDKN2A, exerted a suppressive effect on the tumor immune microenvironment. Consequently, patients in group A had relatively poorer prognoses, while those in group B demonstrated comparatively better outcomes.

### 3.2 Cuproptosis isoforms have different biological pathway activities

**Figure 2:**
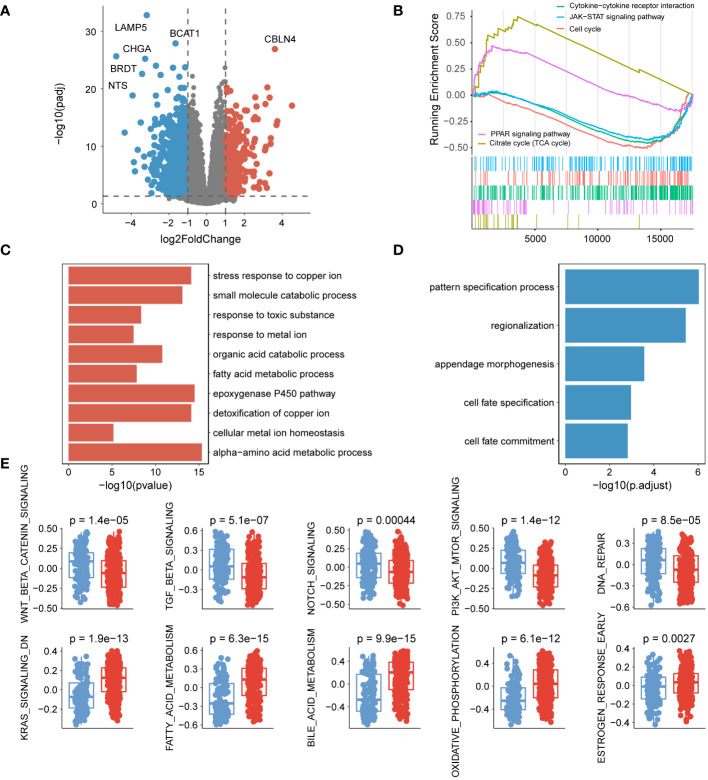
Pathways associated with cuproptosis subtypes. **(A)** Volcano plot displaying the differential expression of genes between the two subtypes. **(B)** GSEA analysis illustrating the enrichment of differentially expressed genes. **(C)** Functional enrichment analysis of up-regulated genes. **(D)** Functional enrichment analysis within different genomic regions of down-regulated genes. **(E)** Comparison of hallmark activity among different isoforms.

To investigate the variations in biological pathways among the different subtypes, differential expression analysis was initially conducted. At a threshold of ploidy change greater than 2, and after correcting for *p*-values less than 0.05, a total of 381 genes were up-regulated, while 857 genes were down-regulated in group B compared to group A (**Figure 2A**, **Supplementary Table 2**). GSEA analysis revealed that PPAR signaling pathway, citrate cycle (TCA cycle), cytokine-cytokine receptor interaction, JAK-STAT, and cell cycle were associated with regulation in the subtypes. Specifically, genes involved in the PPAR signaling pathway and citrate cycle (TCA cycle) were predominantly up-regulated, whereas genes in other pathways were mainly down-regulated (**Figure 2B**). Gene function enrichment analysis showed that the upregulated genes were primarily enriched in the metabolism of metal ions and fatty acids (**Figure 2C**). On the other hand, the downregulated genes were mainly associated with pathways determining cell fate (**Figure 2D**). Furthermore, a hallmark differential analysis confirmed that subtype A exhibited stronger activity in Wnt, TGF_β, Notch, and other signaling pathways. Conversely, subtype B demonstrated increased levels of metabolic responses, specifically involving KRAS and fatty acids (**Figure 2E**).

### 3.3 Identification of key modules associated with immunity

**Figure 3:**
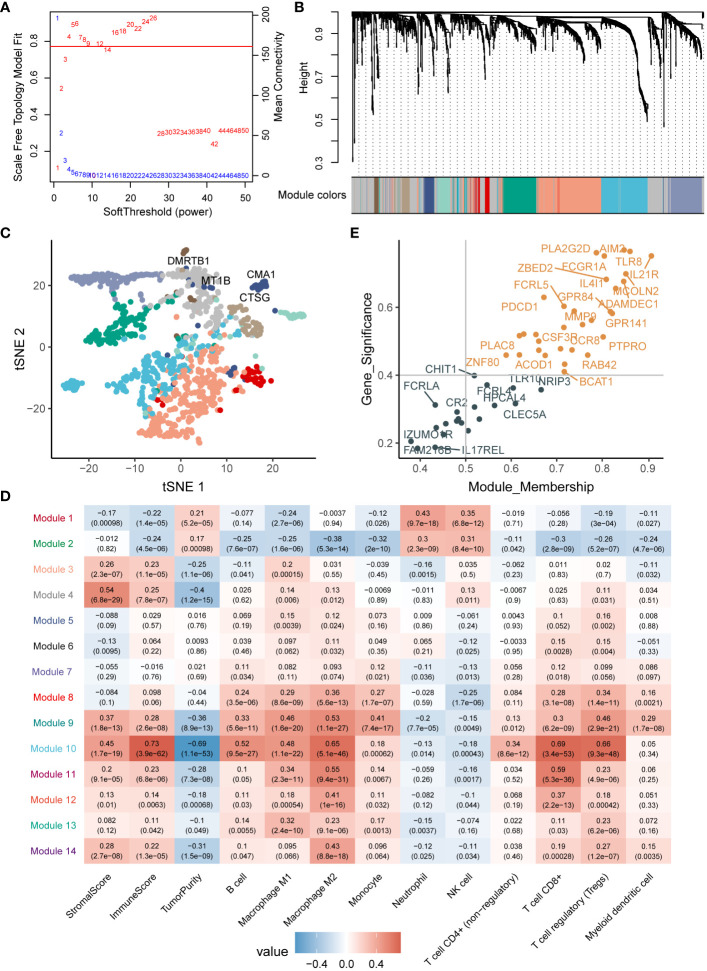
WGCNA analysis of differentially expressed genes. **(A)** Parameter selection for WGCNA analysis. **(B)** Gene modules obtained from WGCNA. **(C)** Validation of differentially expressed genes through downscaled analysis, with dot colors representing the modules. **(D)** Association of modules with the immune microenvironment. **(E)** Identification of key genes within the modules.

To examine the correlation among differentially expressed genes, we utilized the Weighted Gene Co-expression Network Analysis (WGCNA) on TCGA-LIHC dataset, employing a soft threshold (β=4) to construct co-expression modules (**Figure 3A**). The dynamic tree-cut method identified a total of 14 modules (**Figure 3B**, **Supplementary Table 3**), and these modules were depicted to reduce dimensionality (**Figure 3C**). By analyzing module properties through a heatmap, Module 10 was selected as the key module based on immunoscore, immune-cell proportion, and CD8^+^ T-cell infiltration (**Figure 3D**). In Module 10, a total of 31 key genes were identified by integrating module membership and gene significance (**Figure 3E**, **Supplementary Table 3**). This selection proved beneficial for a more in-depth exploration of the relationship between cuproptosis and the tumor immune microenvironment.

### 3.4 Different mutation profiles of cuproptosis isoforms reveal different immunogenicity

**Figure 4:**
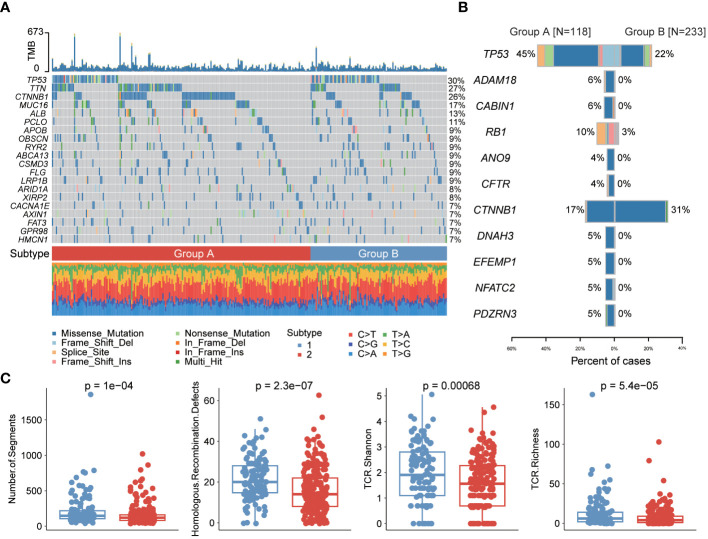
Genomic differences between the two cuproptosis subtypes. **(A)** Mutation profile of liver cancer samples. **(B)** Differences in mutation frequencies between the two subtype samples. **(C)** Differences in immunogenicity between the two subtype samples.

We first showed the mutation profiles of the two subtypes, where the most severely mutated genes included TP53, TTN and MUC16 (**Figure 4A**, **Supplementary Figure S4**). Then, we compared the differences in mutation frequencies between the two subtype samples. The results showed that most of the mutation frequencies in group A were significantly higher than those in group B, such as TP53, ADAM18, CABIN1 and RB1, while only the mutation frequency of CTNNB1 gene was significantly higher in group B than in group A (**Figure 4B**). The large number of mutations in oncogenes may be closely related to cuproptosis and one of the reasons for the poor prognosis of patients in group A. We then compared the immunogenicity differences between the two subtypes, and we found that subtype A samples had higher number of segments, homologous recombination defects and TCR abundance, indicating that subtype A patients are more suitable for immunotherapy (**Figure 4C**).

### 3.5 The cuproptosis score as a prognostic predictor in HCC patients

**Figure 5:**
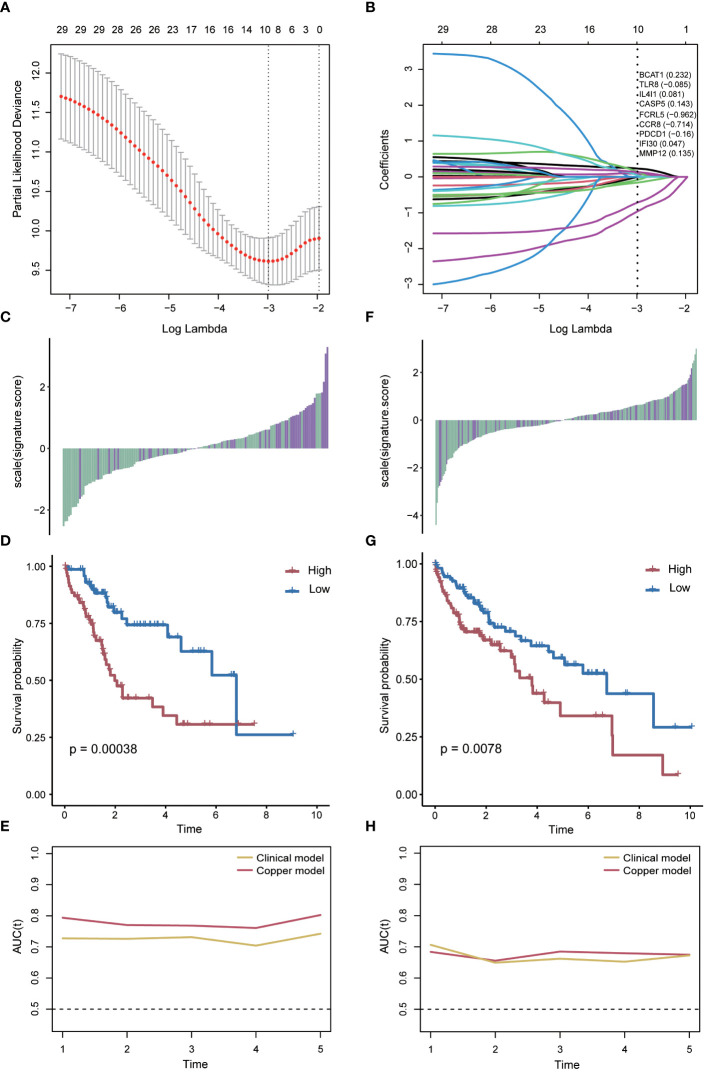
Risk score model based on key genes. **(A)** Parameter screening using Cox-Lasso regression. **(B)** Gene coefficients within the risk scores. **(C)** Relationship between the risk score and patient survival status in the training group. **(D)** Difference in survival time between the high-risk score and the low-risk score groups in the training group. **(E)** AUC values of risk scores predicting survival in the training group. **(F)** Relationship between the risk score and patient survival status in the validation group. **(G)** Difference in survival time between high and low risk score groups in the validation group. **(H)** AUC values of the risk scores predicting survival in the validation group.

Subsequently, a scoring model was developed to assist in predicting the prognosis of hepatocellular carcinoma patients based on the identified key genes associated with cuproptosis and immune response. The liver cancer samples were randomly divided into training and validation sets. Using the LASSO Cox model, we determined a risk scoring system comprising nine genes (BCAT1, TLR8, IL4I1, CASP5, FCRL5, CCR8, PDCD1, IFI30, MMP12) by constraining the training set (**Figures 5A, B**) with the coefficients presented in **Figure 5B**. Furthermore, considering the close relationship between tumorigenesis and methylation (27), we validated the methylation patterns in the UALCAN database (**Supplementary Figure S5**). We then evaluated the prognostic impact of the risk score in the training set by classifying patients into two groups based on the median risk score. The results demonstrated that patients with lower risk scores generally exhibited better survival outcomes compared to those with higher risk scores (*P*=0.00038, log-rank test; **Figures 5C, D**). Moreover, the risk score displayed excellent predictive accuracy at different follow-up times, as indicated by ROC analysis (**Figure 5E**).

To verify that the risk scores retained similar prognostic value across different populations, we applied them to the validation set. Notably, the distribution of risk scores and survival status varied in the validation set (**Figure 5F**). A significant difference in survival time was observed between the high-risk and low-risk groups (*P*=0.0078, log-rank test; **Figure 5G**). The risk score also exhibited outstanding predictive accuracy when assessed in an independently validated cohort (**Figure 5H**).

**Figure 6:**
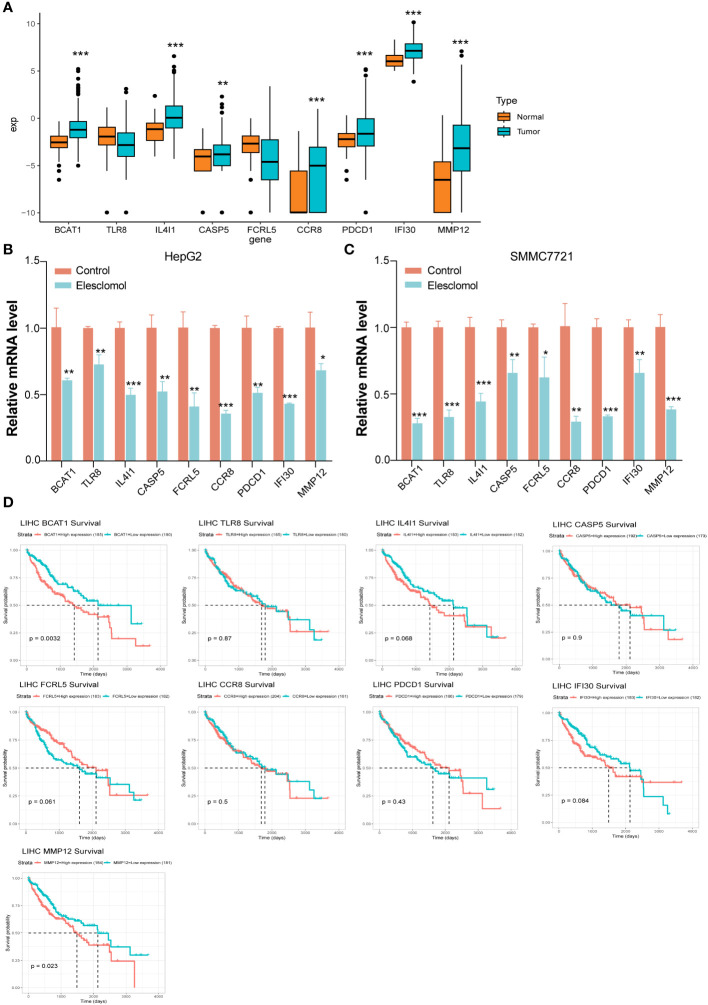
Key genes for validating risk scoring models. **(A)** The genes expression of *BCAT1*, *IL4I1*, *CASP5*, *CCR8*, *PDCD1*, *IFI30*, and *MMP12* were significantly increased in liver cancer patients in the UCSC Xena database. **(B, C)** RT-qPCR indicated that expression of *BCAT1*, *TLR8*, *IL4I1*, *CASP5*, *FCRL5*, *CCR8*, *PDCD1*, *IFI30*, and *MMP12* were significantly down-regulated after Elesclomol treated with HepG2 and SMMC7721 cells for 24h. Data are presented as mean ± SD, *n*=3. *P< 0.05, **P< 0.01, ***P< 0.001. **(D)** The survival analysis curves of these nine genes.

In comparison with the normal group, the genes expression of *BCAT1*, *IL4I1*, *CASP5*, *CCR8*, *PDCD1*, *IFI30*, and *MMP12* were significantly increased in liver cancer patients in the UCSC Xena database (**Figure 6A**). We confirmed the nine cuproptosis-related genes using RT-qPCR in order to verify our results. The HepG2 and SMMC7721 cells were seeded treated using Elesclomol 200 nM for 24 h (**Figures 6B, C**). In comparison with the control group, the expression of *BCAT1*, *TLR8*, *IL4I1*, *CASP5*, *FCRL5*, *CCR8*, *PDCD1*, *IFI30*, and *MMP12* were significantly down-regulated in Elesclomol treatment group (**Figures 6B, C**).

We downloaded the standardized expression data and clinical information of pancancer from the UCSC Xena database, extracted the information of liver Cancer patients, divided them into Normal and cancer according to the characteristics of ID, and analyzed the differential expression of target genes. According to the median expression of a gene, the patients were divided into high expression group and low expression group, and the survival analysis of the gene in the tumor was performed. According to the median expression of a gene, the patients were divided into high expression group and low expression group, and the survival analysis of the gene in the tumor was performed. The survival analysis curves of these nine genes are shown in **Figure 6D**.

### 3.6 Nomogram development for predicting clinical benefit in hepatocellular carcinoma patients

**Figure 7:**
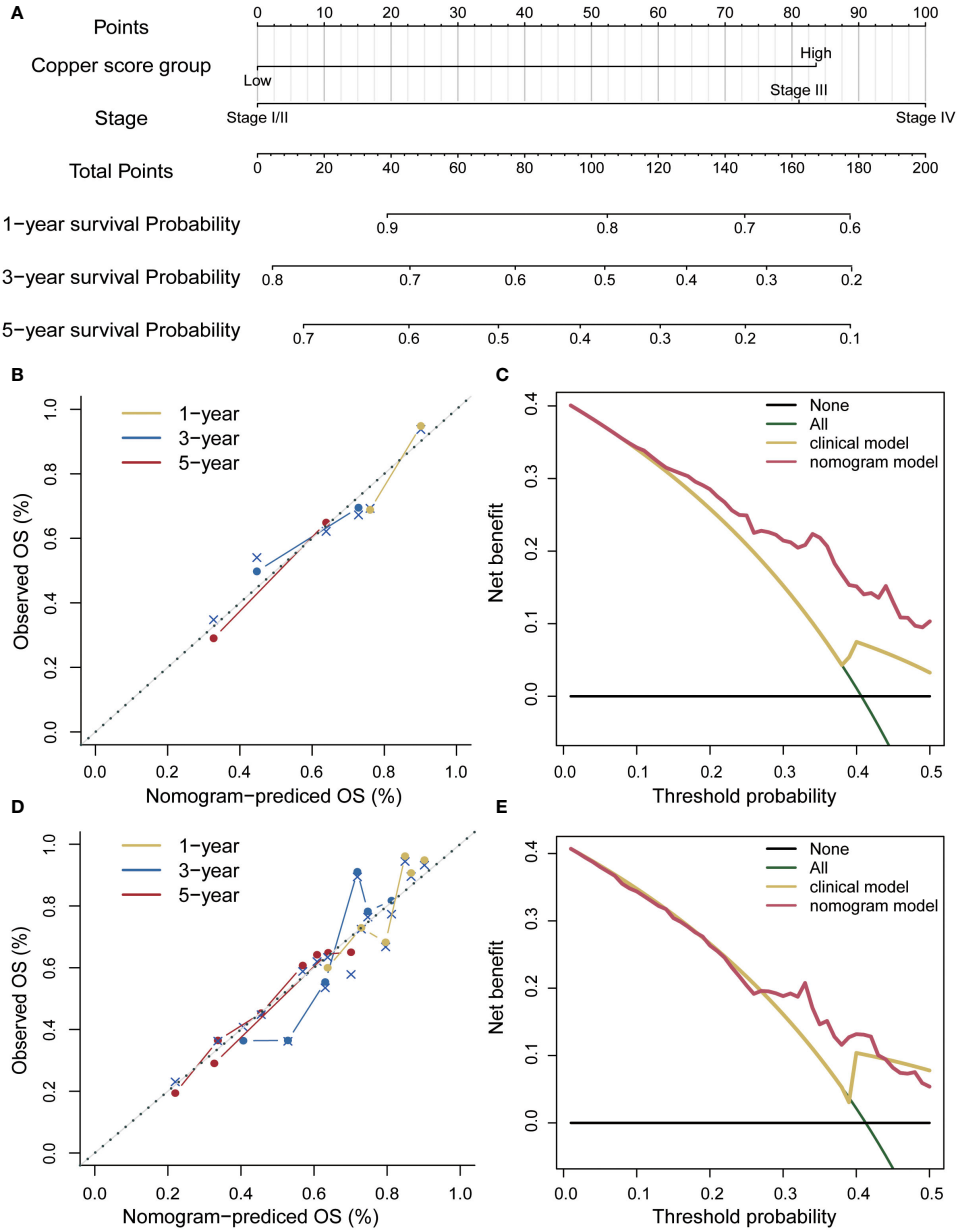
Risk score nomogram. **(A)** Nomogram based on the risk score, age and stage. **(B)** Calibration curves of the column line plot in the training set. **(C)** Decision curve analysis of the nomogram in the training set. **(D)** Calibration curves for the calibration curves in the validation set. **(E)** Decision curve analysis of the nomogram in the validation set.

A prognostic nomogram was developed using a multifactorial Cox regression model (**Figure 7A**). This model integrated the risk score along with two independent predictors, namely age and grading, to better predict the prognosis of patients with hepatocellular carcinoma. The performance of the nomogram was evaluated in both the training set and the independent validation set, demonstrating its effectiveness in predicting patient survival (**Figures 7B, C**). To assess the clinical benefit of the nomogram model, decision curve analysis was conducted on both the training and independent validation sets. The analysis, using 4-year survival as the endpoint, compared the net clinical benefit of the nomogram with several competing intervention strategies, such as intervention for all, intervention for none, and intervention based on different clinical indicators (**Figures 7D, E**). The decision curve analysis revealed that the nomogram provided greater net clinical benefit compared to the other strategies, indicating its potential as a valuable tool for clinical decision-making.

### 3.7 The involvement of cuproptosis in the invasion of T_memory and macrophage_FCN1^+^ immune cells in primary hepatocellular carcinoma

**Figure 8:**
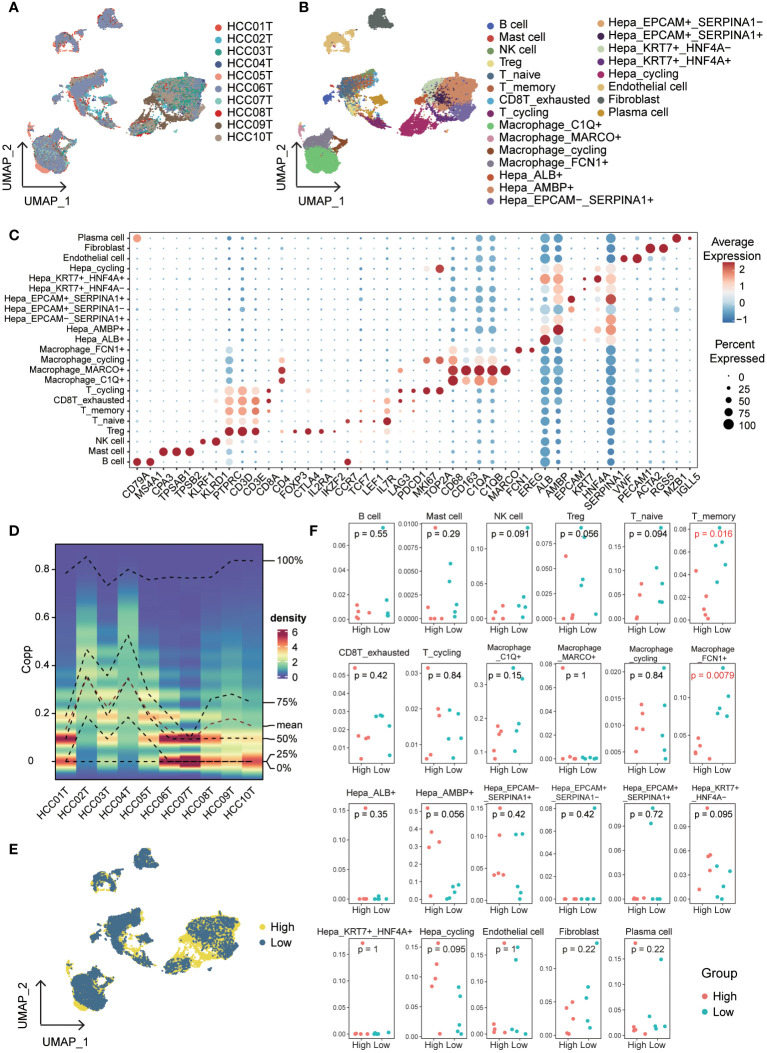
Single-cell overview of primary liver cancer with cuproptosis scores. **(A)** UMAP containing ten different samples. **(B)** UMAP of 23 cell clusters. **(C)** Bubble diagrams of each Cluster’s markers. **(D)** Cuproptosis scores based on the singscore method. **(E)** UMAP of cuproptosis high expression group and cuproptosis low expression group in all cells. **(F)** The proportion of each cluster in cuproptosis high expression group and cuproptosis low expression group.

To explore the impact of cuproptosis on the microenvironment, we analyzed the GSE149614 scRNA-seq dataset for primary hepatocellular carcinoma. **Figure 8A** shows the patient origin of each sample, which was further classified into 23 cell types (**Figure 8B**, **Supplementary Table 5**). We show the markers of clusters (**Figure 8C**, **Supplementary Figure S6**) and the distribution and proportion of immune and non-immune cells in them (**Supplementary Figures S7A, B**). We examined the expression of cuproptosis-related genes within each cell cluster and found that these genes were predominantly expressed at higher levels in non-immune cells (**Supplementary Figure S7C**). To assess the variability in gene expression, we used the singscore method and observed distinct expression patterns among samples and clusters (**Figure 8D**, **Supplementary Table 6**). Consequently, we divided the samples into two groups based on high and low expression of cuproptosis-related genes, equally distributed (**Supplementary Figure S7D**). The expression and distribution of cuproptosis-related genes were then analyzed across different cell subpopulations (**Figure 8E**). Furthermore, we compared the proportions of immune cells in the high and low cuproptosis-related gene expression groups. Notably, T_memory and Macrophage_FCN1^+^ were significantly lower in the high-expression group compared to the low-expression group (**Figure 8F**, **Supplementary Table 7**). Previous studies have highlighted the role of FCN^+^ macrophages in recognizing and eliminating tumor cells by detecting abnormal glycosylation patterns on their surface (28, 29). Additionally, the infiltration of T memory cells within tumors allows for quicker and stronger immune responses (30). These findings suggest that a low-score cuproptosis environment can activate the immune system, promote inflammation, and facilitate the recruitment of immune cells, thereby enhancing the ability to attack tumor cells.

### 3.8 The cell-cell communication among T_memory cells exhibit significant variation based on the expression levels of cuproptosis-related genes

**Figure 9:**
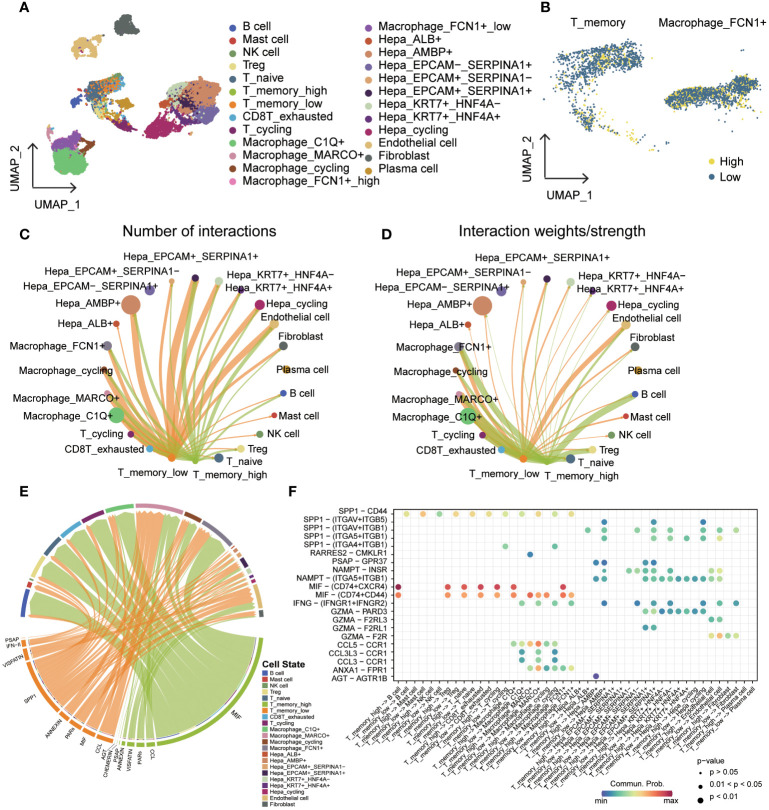
Cell-cell communication in groups with different cuproptosis-related gene expression levels. **(A)** UMAP of cell types added to the cuproptosis group. **(B)** Cuproptosis groups of T_memory and Macrophage_FCN1^+^. **(C)** T_memory_high and T_memory_low are presented as a cell-cell communication network of the signaling sender with all other cells, with a circle diagram showing the numbers of interactions between any two cell groups. **(D)** T_memory_high and T_memory_low are presented as a cell-cell communication network of the signaling sender with all other cells, with a circle diagram showing the interaction weights/strength between any two cell groups. **(E)** Chord diagram showing the difference between the signals sent by T_memory_high and T_memory_low. **(F)** Bubble diagram showing ligand-receptor mediated cellular interactions when cells act as signal transmitters.

Based on the expression levels of cuproptosis-related genes, we categorized the T_memory and Macrophage_FCN1^+^ cells into high and low expression groups (**Figures 9A, B**). Our investigation focused on understanding the differences in cell-cell communication and signaling between these groups, with a goal of identifying the key factors contributing to phenotypic variation. Notably, we found no significant differences in the number of interactions and interaction weights/strength between Macrophage_FCN1^+^ cells, regardless of whether they acted as signal senders or receivers (**Supplementary Figures S8A–D**). Similar results were observed when T_memory cells were the signal receivers (**Supplementary Figures S8E, F**). However, we observed distinct patterns when T_memory cells were the signal senders. Specifically, T_memory_low cells emitted a higher number of signals compared to T_memory_high cells. Additionally, in terms of signal intensity, T_memory_low cells generally exhibited stronger signaling towards non-immune cells, while T_memory_high cells displayed stronger signaling towards immune cells (**Figures 9C, D**).

In our further analysis, we investigated the signaling pathways involved in T_memory_high and T_memory_low cells. Notably, T_memory_high cells displayed stronger MIF signals towards almost all other cells, while T_memory_low cells predominantly sent SPP1 signals (**Figure 9E**, **Supplementary Figure S9A**). Furthermore, we identified receptor-ligand pairs that played a pivotal role in these signaling pathways. T_memory_low cells communicated SPP1/CD44 signals to all other immune cells, indicating a high degree of immune infiltration. Additionally, non-immune cells, such as SPP1-(ITGAV+ITGB5), received SPP1 signals. The IFNG pathway exhibited significant variation, primarily observed in macrophages and a subset of hepatocytes, with the main receptor-ligand pair being INFG-(IFNGR1+IFNGR2). Macrophages also released CCL signals. On the other hand, the MIF signaling pathway was predominantly enriched in T_memory_high cells and relied on MIF-(CD74+CXCR4) and MIF-(CD74+CD44) interactions. This signaling cascade was responsible for communication with T cells, B cells, and macrophages (**Figure 9F**, **Supplementary Figure S9B**).

### 3.9 Knockdown of DLAT and CDKN2A shows tumor−suppressive effects

**Figure 10:**
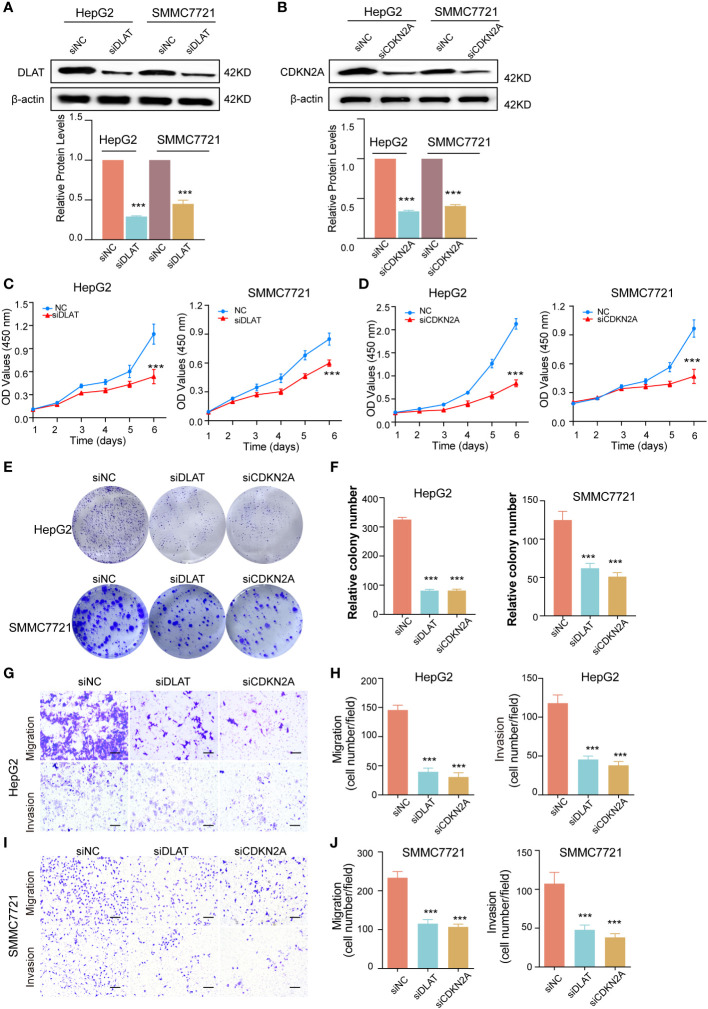
The effect of knockdown of DLAT and CDKN2A on cell proliferation, migration and invasion. **(A)** Western blot indicated that DLAT protein expression levels were decreased after knockdown of DLAT in HepG2 and SMMC7721 cells (above). Bands were quantified with Image J software and evident from the quantification of Histogram. β-actin was used as a loading control. Data are presented as mean ± SD, *n*=3. ***P< 0.001 (down). **(B)** Western blot indicated that CDKN2A protein expression levels were decreased after knockdown of CDKN2A in HepG2 and SMMC7721 cells. (above). Bands were quantified with Image J software and evident from the quantification of Histogram. β-actin was used as a loading control. Data are presented as mean ± SD, *n*=3. ***P< 0.001 (down). **(C, D)** CCK-8 assay showed that cells proliferation was inhibited. Colony formation assays, with representative images **(E)** and bar graphs **(F)** from three independent experiments, demonstrated that cells proliferation was inhibited in cells with DLAT and CDKN2A knockdown. Data expressed as mean ± SD. ***P< 0.001. **(G-J)** Comparison of the migration and invasion of HepG2 and SMMC7721 cells using transwell compartments. Representative images **(G, I)** and statistics **(H, J)** of migration and invasion assay were shown.

We knocked down DLAT and CDKN2A expression by using siRNAs in HepG2 and SMMC7721 cells, which was verified by western blot (**Figures 10A, B**). CCK-8 and colony formation assays were used to evaluate the proliferation of HepG2 and SMMC7721 cells. We found that DLAT and CDKN2A knockdown significantly inhibited cell proliferation (**Figures 10C, D**). Cells of different treatment groups were inoculated into 6-wellplates. The plates were seeded with 2000 cells (HepG2) or 2500 cells (SMMC7721). The cells were fixed after 14 days of culture, stained with crystal violet and then counted. The results showed that the colony formation rate was significantly lower in the DLAT and CDKN2A knockdown group than in the control groups (**Figures 10E, F**). Our studies indicated that inhibition of DLAT and CDKN2A expression significantly reduced the proliferation of liver cancer cells. The effect of knocked down DLAT and CDKN2A on the *in vitro* tumor cell migration and invasion was investigated using the Transwell migration and Matrigel invasion assay, respectively. Under light microscope, the crystal violet-stained HepG2 and SMMC7721 cells demonstrated reduced motility after knocked down DLAT and CDKN2A (**Figures 10G–J**).

## 4 Discussion

The role of cuproptosis, a newly discovered regulated cell death mechanism, in cancer is not yet fully understood (31). In this study, we conducted a systematic analysis to explore the relationship between cuproptosis and the immune microenvironment in hepatocellular carcinoma, and we identified three key findings: (1) We developed a cuproptosis-related prognostic model, which was validated as a reliable tool for predicting clinical patient outcomes. (2) Using the WGCNA algorithm, we identified key immune-related gene modules among differentially expressed genes with distinct cuproptosis patterns. This analysis allowed us to establish a risk scoring system consisting of 9 genes. (3) At the single-cell level, we identified cell populations associated with cuproptosis. We divided the samples into high and low cuproptosis groups based on their respective scores and compared the differences in cell-to-cell communication.

In hepatocellular carcinoma, we observed two distinct patterns of cuproptosis regulation. One subtype exhibited high expression of three cuproptosis negatively regulated genes (DLAT, GLS, and CDKN2A), which was associated with a lower survival rate. This subtype showed increased activation of the Wnt signaling pathway, and recent studies have highlighted the link between cuproptosis and Wnt signaling pathway (32). Furthermore, the TGF-β pathway was found to be highly active in this subtype. TGF-β acts as an immunosuppressive cytokine and exerts a broad suppressive effect on the immune response through various mechanisms (33). In early-stage tumors, the TGF-β pathway induces apoptosis and inhibits tumor cell proliferation (34). Previous research has also indicated a correlation between DLAT and programmed death ligand 1 (PD-L1) (35). Differences in GLS expression may influence cancer outcomes in humans (36). CDKN2A, known as multiple tumor suppressor 1 (MTS1), is a biomarker for immunoinfiltration in multiple cancers (37). Our study sheds light on the relationship between cuproptosis and the immune microenvironment in hepatocellular carcinoma, providing valuable insights into potential prognostic markers and cellular pathways involved in this context.

The other subtype, characterized by high expression of FDX1, MTF1, LIAS, and LIPT1, exhibits elevated levels of cuprotosis positive regulatory genes and a better prognosis. One of these genes, FDX1, plays a crucial role in regulating Cu carrier-induced cell death by converting Cu^2+^ into a more toxic form within cells (9). MTF1, known as metal regulatory transcription factor 1, regulates the expression of metallothioneins (MTs) and is closely associated with the excretion of Cu and other metals (38). Recent studies propose that the FDX1-LIAS axis, which consists of Ferredoxin 1 and Lipoic Acid Synthetase, serves as a key signaling pathway in cuprotosis. This pathway plays a significant role in regulating cellular oxidative stress and directly influences cell survival by inducing or promoting oxidative stress (39). Furthermore, the knockdown of the LIPT1 gene has been shown to inhibit the proliferation and invasion of hepatocellular carcinoma cells. This finding suggests that LIPT1 may promote the proliferation, invasion, and migration of hepatocellular carcinoma cells (LIHC) (40). Additionally, this isoform demonstrates active KRAS signaling and plays a role in fatty acid and bile acid metabolism. This indicates that this particular isoform possesses enhanced hepatocellular functions and exhibits stronger responses in terms of oxidative stress and metabolism. In summary, the subtype characterized by high expression of FDX1, MTF1, LIAS, and LIPT1 represents a distinct group in relation to cuprotosis regulation. Understanding the functions and pathways associated with this subtype provides valuable insights into the molecular mechanisms underlying hepatocellular carcinoma and its response to oxidative stress and metabolic alterations.

Using the WGCNA algorithm, we discovered multiple modules comprised of differentially expressed genes exhibiting diverse protrusion patterns. Among these modules, we focused on those highly relevant to immunity. Notably, the key gene PDCD1 serves as an immune checkpoint (41). Additionally, we identified CSF3R and FCGR1A as myeloid markers (42, 43), IL21R and IL4I1 as cytokines and chemokines acting as immune modulators (44, 45).These findings suggest that the regulation of cuproptosis can reshape the tumor microenvironment (TME) and consequently impact the efficacy of immunotherapy. Furthermore, we developed a scoring model comprising nine key genes (BCAT1, TLR8, IL4I1, CASP5, FCRL5, CCR8, PDCD1, IFI30, MMP12) associated with cuproptosis and TME. The validation set confirmed the model’s favorable prognostic effect. RT-qPCR was conducted to validate our findings, which were consistent with results obtained from bioinformatics tools, thereby reaffirming the significant role of cuproptosis-related biomarkers in HCC. BCAT1 is closely associated with autophagy in cancer cells (46). The TLR8-MyD88 signaling pathway regulates antigen specificity and the inhibitory function of CD4 Treg cells (47). CASP5 is intricately involved in copper metabolism, apoptosis, and autophagy (48). FCRL5 belongs to the Ig superfamily of molecules known as Fc receptor-like (FCRL), exhibiting tyrosine-based immunomodulatory potential while being closely linked to B cell signaling (49). IFI30 has been reported to be significantly and positively associated with immune checkpoints that impede effective anti-tumor immune responses (50). MMP12 is highly correlated with a poor prognosis in hepatocellular carcinoma (51).

scRNA-seq has emerged as an invaluable tool for classifying cell types based on transcriptional profiles in various cancer types. In this study, we obtained single-cell data for primary liver cancer from the GEO database and performed specific marker identification to assign these cells into 23 distinct cell types. Notably, we observed similar expression patterns of cuproptosis-associated genes across these cell types. To quantitatively assess the expression of cuproptosis, we applied singscore, resulting in the division of all samples into high and low expression groups (1:1) based on their cuproptosis scores. This categorization allowed us to make comparisons between different cell types. We observed a significant difference between T_memory cells and Macrophage_FCN1^+^ cells, indicating the involvement of cuproptosis in reshaping the tumor immune microenvironment in hepatocellular carcinoma. Specifically, we noticed variations in the infiltration of memory T cells and TAM (tumor-associated macrophages). Interestingly, cells with low cuproptosis scores, including T_memory cells and Macrophage_FCN1^+^ cells, were found to generate an activated immune microenvironment, effectively exerting anti-tumor functions. Consequently, patients with hepatocellular carcinoma exhibiting this immune microenvironment experienced a more favorable prognosis. Moreover, these findings have implications for the response of hepatocellular carcinoma patients to immunotherapy, highlighting the potential for cuproptosis regulation to influence treatment outcomes.

Finally, we examined the differences in cell-cell communication between memory T cells and TAM cells in relation to cuproptosis. Notably, we observed distinct signaling patterns in the cuproptosis-related cell communication of T memory cells, which may be closely associated with tumor development. Further analysis revealed that T_memory_high cells predominantly exhibited stronger MIF signals, while T_memory_low cells displayed higher levels of SPP1 signals. In terms of specific ligand-receptor interactions, we found that SPP1/CD44 mediated the interactions of T_memory cells with other immune cells, whereas other ligands of SPP1 were involved in interactions with non-immune cells. SPP1/CD44 is commonly known to facilitate the interaction between cancer cells and TAM, thereby promoting cancer progression (52). Our findings indicate diverse immune cell interactions that may impact T_memory infiltration and immunotherapy. Furthermore, we observed higher levels of CCL3/CCR1 (53) and IFNG-(IFNGR1+IFNGR2) (54) in T_memory_low cells, which are associated with a favorable tumor prognosis. In contrast, another subset of T_memory cells representing samples with a high incidence of cuproptosis exhibited a strong MIF signaling interaction with immune cells. Notably, MIF-(CD74+CXCR4) (55) played a prominent role in this interaction, promoting tumorigenesis through multiple mechanisms.

To further verify the role of DLAT and CDKN2A in HCC, we performed *in vitro* experiments, including CCK-8, cell colony formation, migration, and invasion assays. The results of *in vitro* experiments showed that when DLAT or CDKN2A was knocked down, the proliferation, migration, and invasion of HCC cells were significantly decreased. However, the study still has some limitations. Most of our results are limited to data mining analysis results, and we need more *in vitro* and *in vivo* experiments to investigate the exact mechanism of DLAT or CDKN2A in LIHC.

While our study provided crucial insights into the association between cuproptosis and the tumor microenvironment (TME) through the analysis of a large number of tumor samples, the experimental validation and large-scale clinical trials are necessary to confirm these findings. This stands as a notable limitation of our study, but it also highlights the need for future research in this area. In conclusion, our study sheds light on the close relationship between cuproptosis and TME, and the integration of cuproptosis patterns holds promise for optimizing immunotherapy strategies for patients diagnosed with hepatocellular carcinoma.

## Data availability statement

The datasets presented in this study can be found in online repositories. The names of the repository/repositories and accession number(s) can be found in the article/**Supplementary Material**.

## Ethics statement

Ethical approval was not required for the studies on humans in accordance with the local legislation and institutional requirements because only commercially available established cell lines were used. Ethical approval was not required for the studies on animals in accordance with the local legislation and institutional requirements because only commercially available established cell lines were used.

## Author contributions

YW: Conceptualization, Data curation, Funding acquisition, Methodology, Supervision, Validation, Writing – original draft, Writing – review & editing. XG: Data curation, Methodology, Writing – review & editing. XM: Conceptualization, Data curation, Formal analysis, Methodology, Supervision, Validation, Writing – original draft. JP: Conceptualization, Data curation, Methodology, Supervision, Writing – review & editing.
