# Supplementary material for: Distinct cuproptosis patterns in hepatocellular carcinoma patients correlate with unique immune microenvironment characteristics and cell-cell communication, contributing to varied overall survival outcomes
Source: Front Immunol. 2024 May 28;15:1379690. doi: 10.3389/fimmu.2024.1379690 (PMC11168106; doi:10.3389/fimmu.2024.1379690)
Supplement: Supplementary file 1 [file Image_1.pdf]

Supplementary Figures

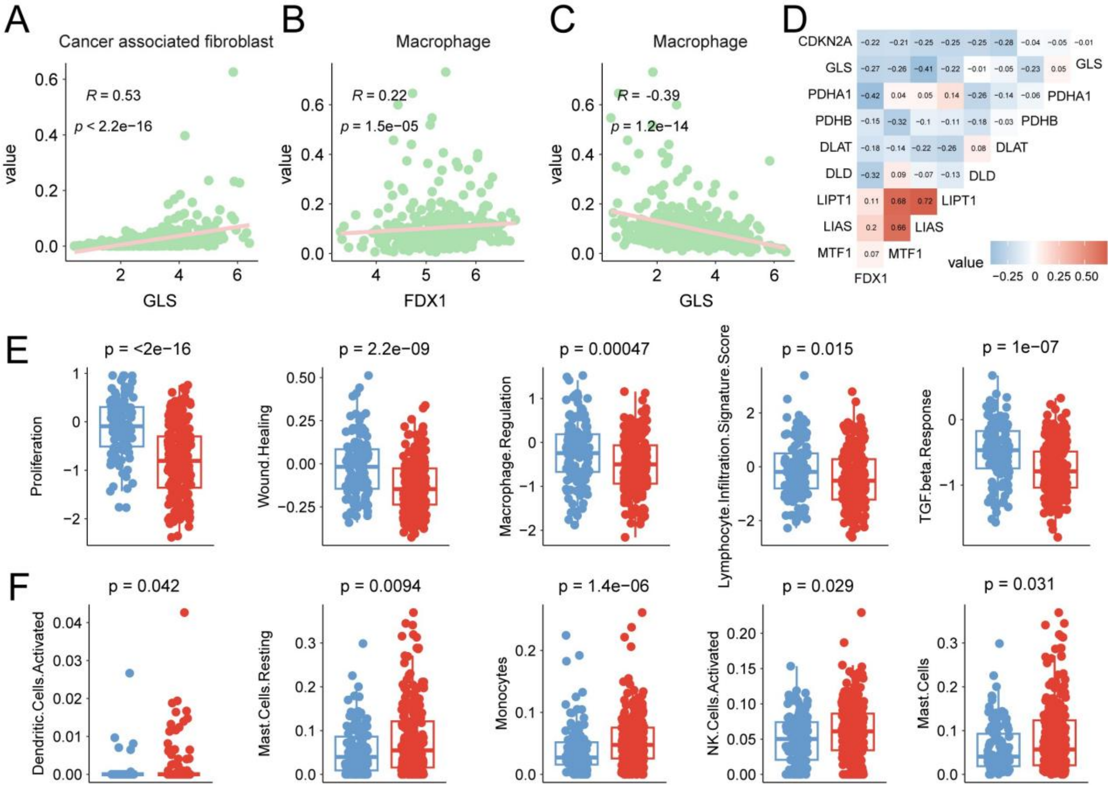

Figure S1. Association of cuproptosis-related genes and immune microenvironment. (A) Correlation between cuproptosis-related genes and fibroblast. (B) Correlation between FDX1 and macrophages. (C) Correlation between GLS and macrophages. (D) Correlation between genes associated with cuproptosis. (E) Immune microenvironment characteristics of subtype A. (F) Immune microenvironment characteristics of subtype B.

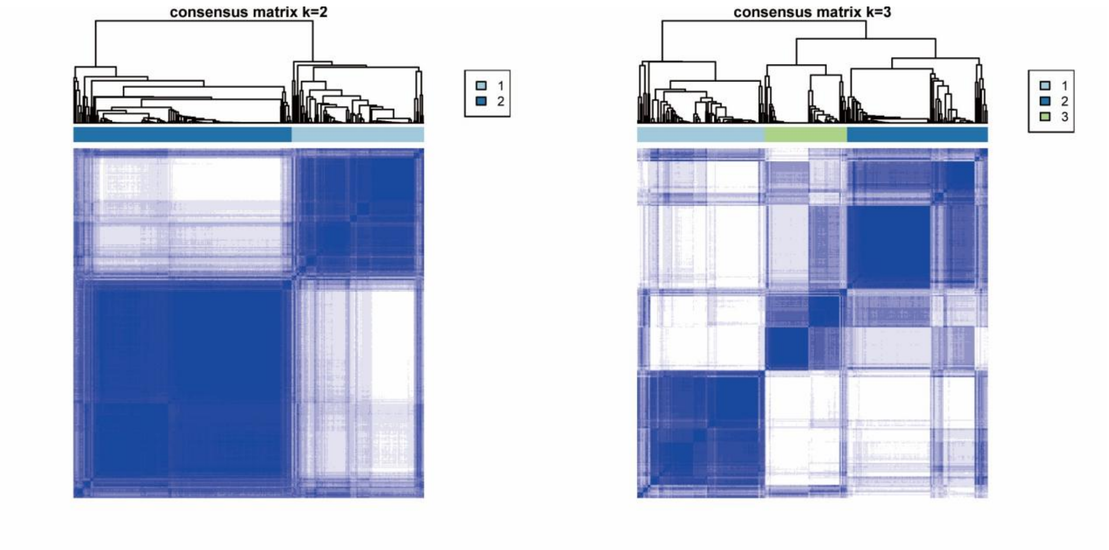

Figure S2. Consensus matrix from consensus clustering.

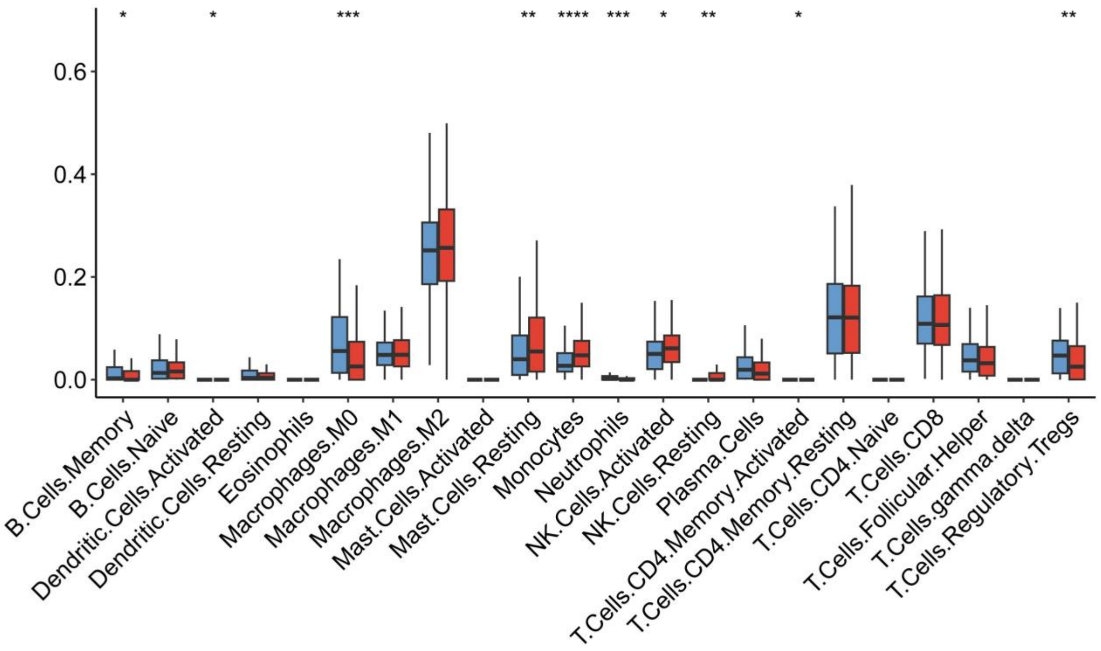

Figure S3. The difference of different cuproptosis subtypes of immune cell infiltration. The proportion of immune cells was determined by CIBERSORT algorithm.

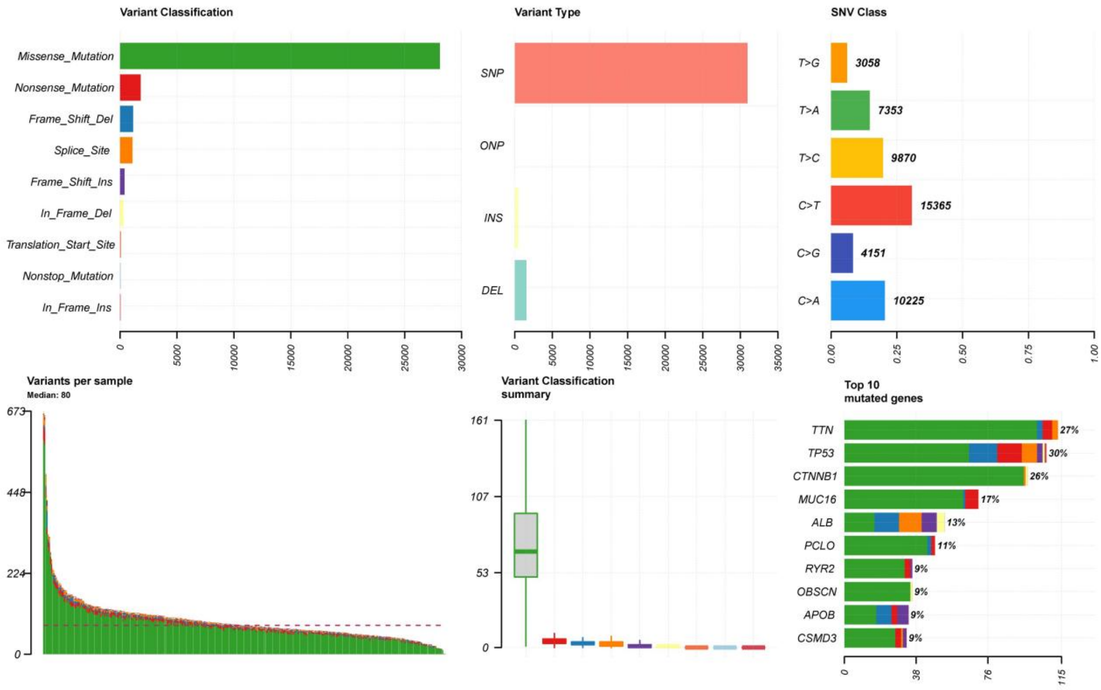

Figure S4. Mutation summary of hepatocellular Carcinoma.

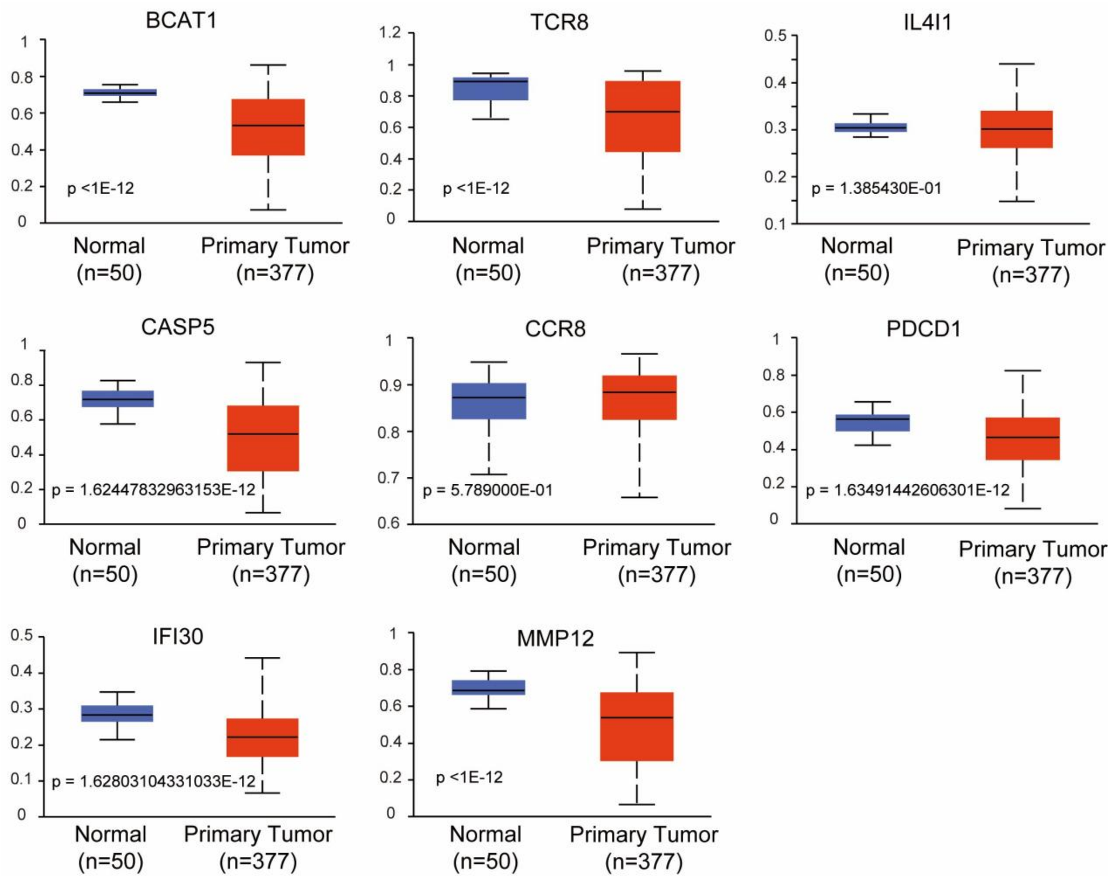

Figure S5. The methylation profiles of 9 genes in HCC and normal subjects were obtained from the UALCAN database. Average Beta value for "FCRL5" is not available for majority of samples in LIHC.

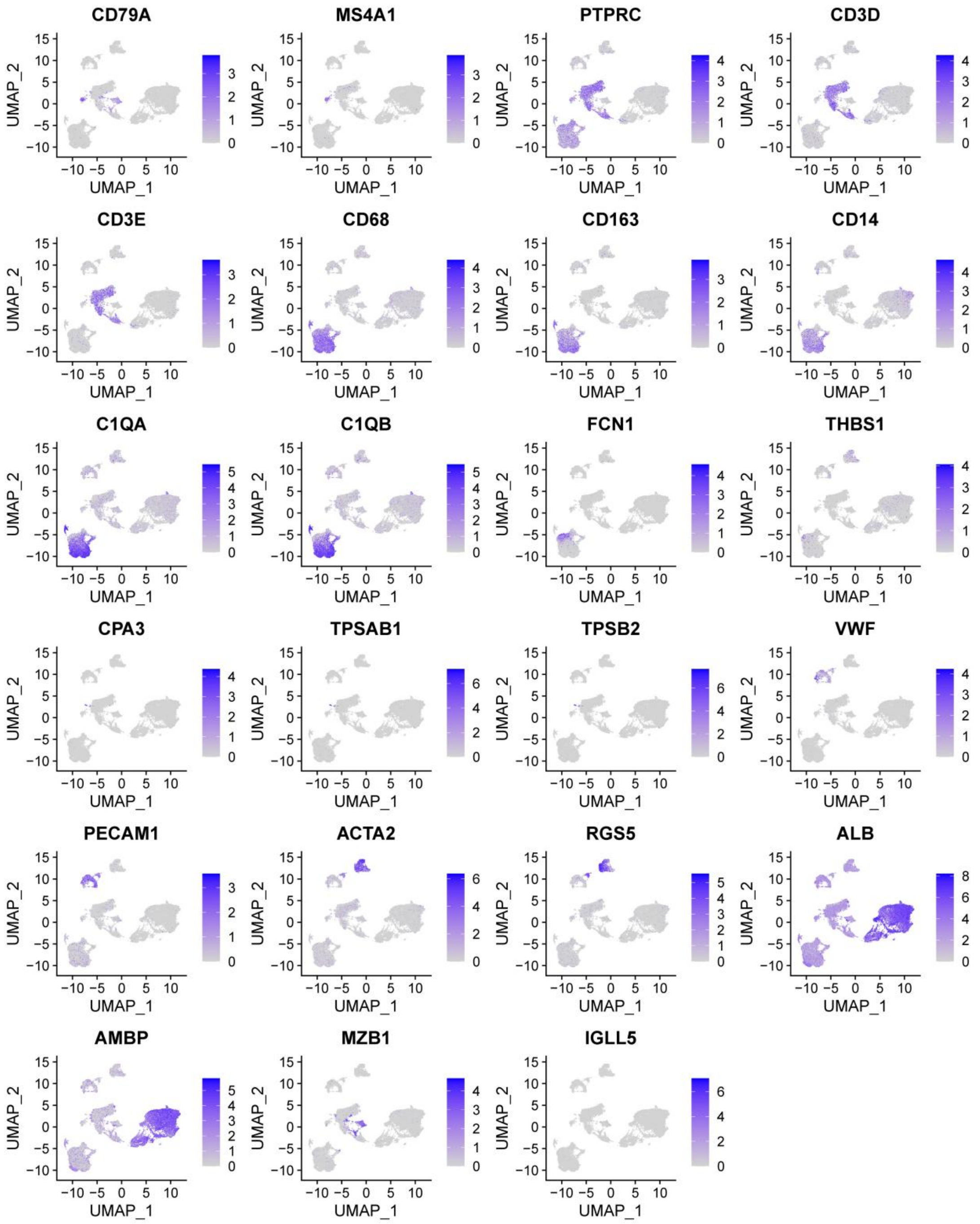

Figure S6. Markers for the 23 clusters.

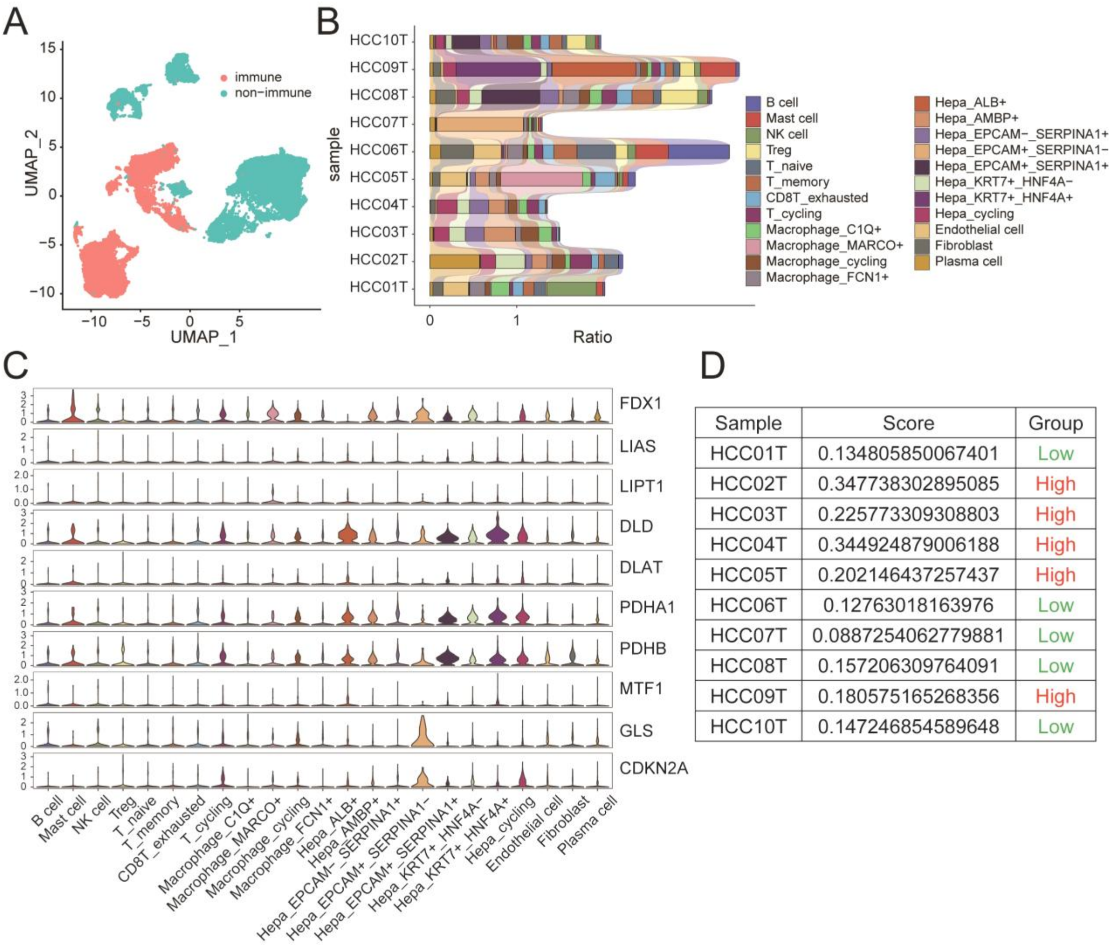

Sample	Score	Group
HCC01T	0.134805850067401	Low
HCC02T	0.347738302895085	High
HCC03T	0.225773309308803	High
HCC04T	0.344924879006188	High
HCC05T	0.202146437257437	High
HCC06T	0.12763018163976	Low
HCC07T	0.0887254062779881	Low
HCC08T	0.157206309764091	Low
HCC09T	0.180575165268356	High
HCC10T	0.147246854589648	Low

Figure S7. Single-cell clusters and cuproptosis scores. (A) Umap of immune and non-immune cells. (B) The proportion of immune and non-immune cells in each sample. (C) Expression of cuproptosis-related genes in each cluster. (D) Cuproptosis scores grouping.

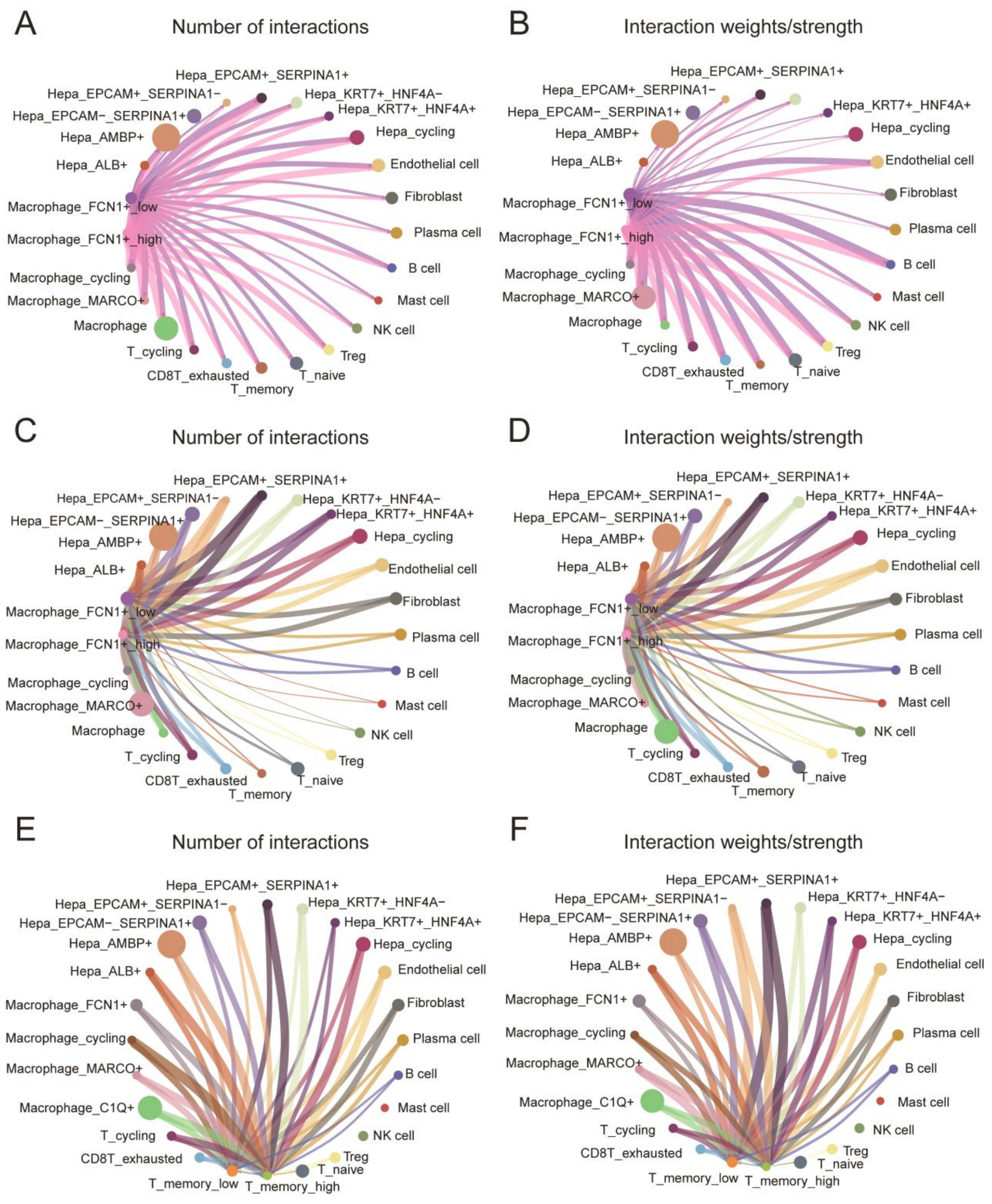

Figure S8. Visualization of cell-cell communication associated with cuproptosis. (A) Macrophage_FCN1$^+$_high and Macrophage_FCN1$^+$_low are presented as a cellular communication network of the signaling sender with all other cells, with a circle diagram showing the numbers of interactions between any two cell groups. (B) Macrophage_FCN1$^+$_high and Macrophage_FCN1$^+$_low are presented as a cellular communication network of the signaling sender with all other cells, with a circle diagram showing the interaction weights/strength between any two cell groups. (C)

Macrophage_FCN1$^+$_high and Macrophage_FCN1$^+$_low are presented as a cellular communication network of the signaling receiver with all other cells, with a circle diagram showing the numbers of interactions between any two cell groups. (D) Macrophage_FCN1$^+$_high and Macrophage_FCN1$^+$_low are presented as a cellular communication network of the signaling receiver with all other cells, with a circle diagram showing the interaction weights/strength between any two cell groups. (E) T_memory_high and T_memory_low are presented as a cellular communication network of the signaling receiver with all other cells, with a circle diagram showing the numbers of interactions between any two cell groups. (F) T_memory_high and T_memory_low are presented as a cellular communication network of the signaling

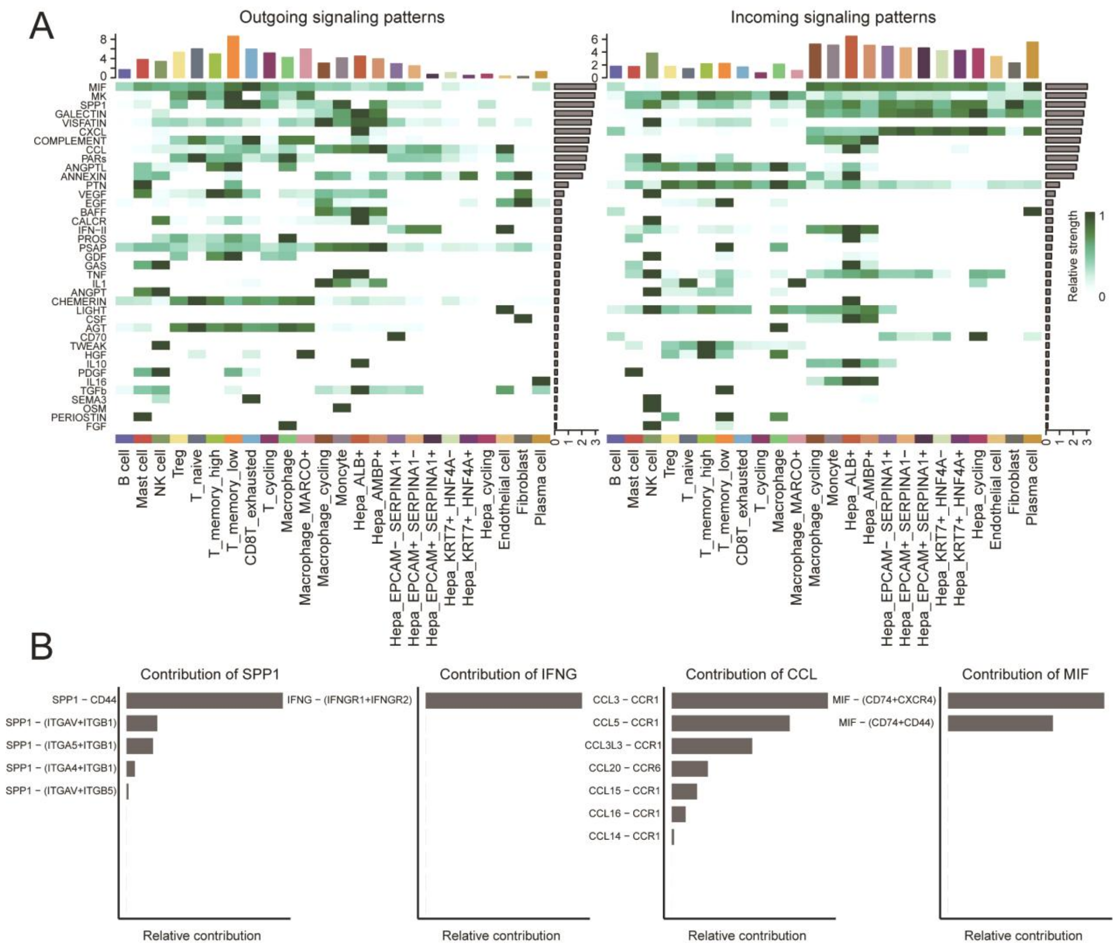

Figure S9. Signal visualization related to cuproptosis. (A) The signal that contributes the most to the outgoing or incoming T_memory_high and T_memory_low signals. (B) The contribution of each ligand-receptor pair to the overall signaling pathway.

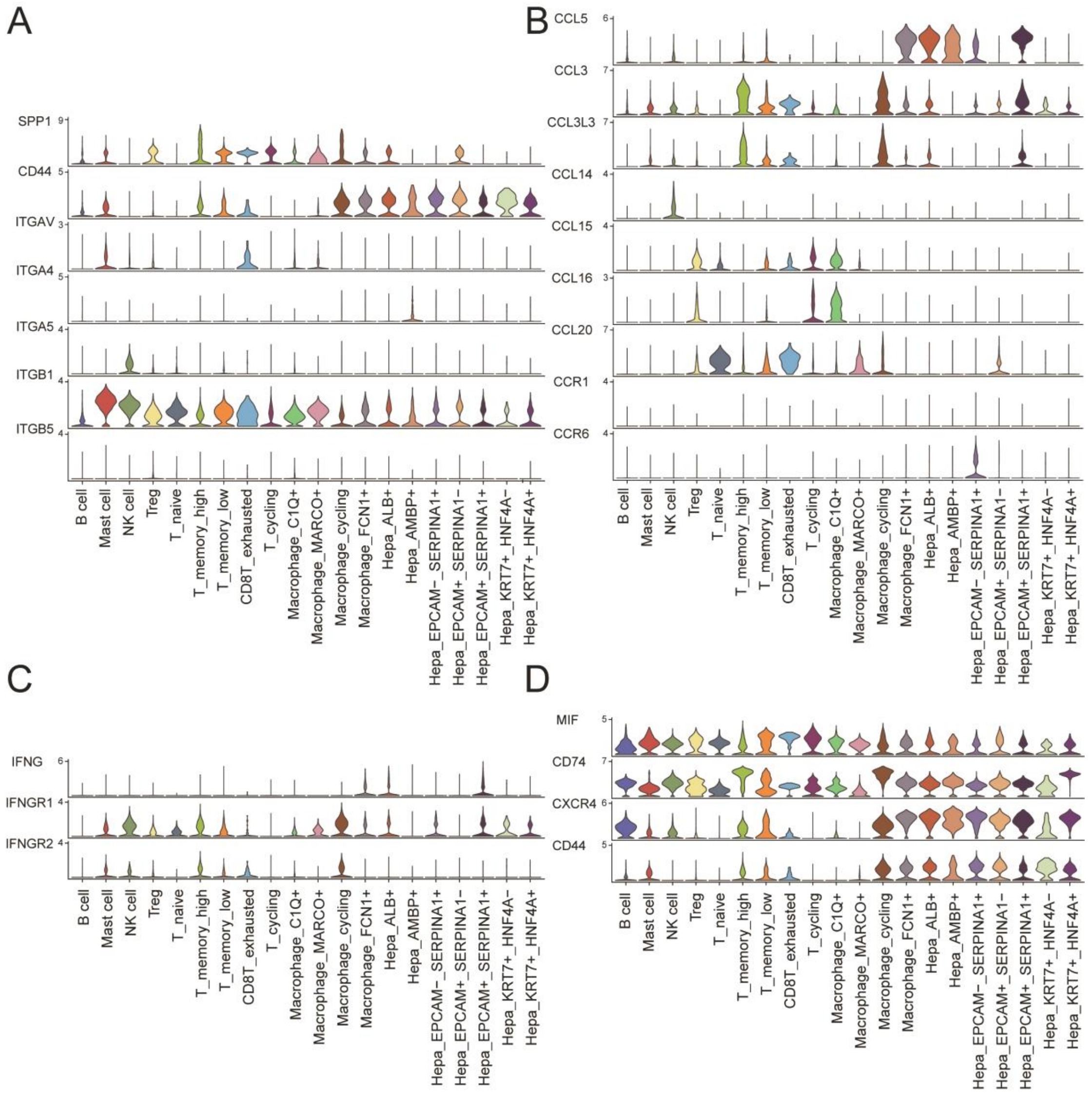

Figure S10. Expression distribution of cuproptosis-related signaling genes. (A) Violin plot of SPP1 signaling gene expression distribution. (B) Violin plot of CCL signaling gene expression distribution. (C) Violin plot of IFNG signaling gene expression distribution. (D) Violin plot of MIF signaling gene expression distribution.

Supplementary Tables

Table S1. Association of cuproptosis subtypes with existing subtypes.

Table S2. Differential expression analysis among cuproptosis subtypes.

Table S3. Gene modules from WGCNA.

Table S4. Cox analysis of cuproptosis score.

Table S5. Markers of Sc-RNAseq data.

Table S6. Scores of cuproptosis.

Table S7. Table S7. Data of figure 7F.
